# A Novel Peptide Derived from the Fusion Protein Heptad Repeat Inhibits Replication of Subacute Sclerosing Panencephalitis Virus *In Vitro* and *In Vivo*

**DOI:** 10.1371/journal.pone.0162823

**Published:** 2016-09-09

**Authors:** Masahiro Watanabe, Koichi Hashimoto, Yusaku Abe, Eiichi N. Kodama, Ryota Nabika, Shinya Oishi, Shinichiro Ohara, Masatoki Sato, Yukihiko Kawasaki, Nobutaka Fujii, Mitsuaki Hosoya

**Affiliations:** 1 Department of Pediatrics, Fukushima Medical University, Fukushima, Japan; 2 Division of Emerging Infectious Diseases, Tohoku University School of Medicine, Sendai, Japan; 3 Graduate School of Pharmaceutical Sciences, Kyoto University, Kyoto, Japan; Deutsches Primatenzentrum GmbH - Leibniz-Institut fur Primatenforschung, GERMANY

## Abstract

Subacute sclerosing panencephalitis (SSPE) is a persistent, progressive, and fatal degenerative disease resulting from persistent measles virus (MV) infection of the central nervous system. Most drugs used to treat SSPE have been reported to have limited effects. Therefore, novel therapeutic strategies are urgently required. The SSPE virus, a variant MV strain, differs virologically from wild-type MV strain. One characteristic of the SSPE virus is its defective production of cell-free virus, which leaves cell-to-cell infection as the major mechanism of viral dissemination. The fusion protein plays an essential role in this cell-to-cell spread. It contains two critical heptad repeat regions that form a six-helix bundle in the trimer similar to most viral fusion proteins. In the case of human immunodeficiency virus type-1 (HIV-1), a synthetic peptide derived from the heptad repeat region of the fusion protein enfuvirtide inhibits viral replication and is clinically approved as an anti-HIV-1 agent. The heptad repeat regions of HIV-1 are structurally and functionally similar to those of the MV fusion protein. We therefore designed novel peptides derived from the fusion protein heptad repeat region of the MV and examined their effects on the measles and SSPE virus replication *in vitro* and *in vivo*. Some of these synthetic novel peptides demonstrated high antiviral activity against both the measles (Edmonston strain) and SSPE (Yamagata-1 strain) viruses at nanomolar concentrations with no cytotoxicity *in vitro*. In particular, intracranial administration of one of the synthetic peptides increased the survival rate from 0% to 67% in an SSPE virus-infected nude mouse model.

## Introduction

The measles virus (MV) is a nonsegmented negative-strand RNA virus that belongs to the family Paramyxoviridae in the genus *Morbillivirus*. MV has six transcription units—N, P, M, F, H, and L genes—which encode nucleocapsid (N), phospho (P), matrix (M), fusion (F), hemagglutinin (H), and large polymerase (L) proteins. Ribonucleoprotein complexes are comprised of the N, P, and L proteins, as well as the viral RNA genome [1–3]. The P locus also encodes nonstructural V and C proteins functioning as virulence factors [4]. The H protein is associated with binding to host cell receptors, such as CD46 [5] and PVRL4 (Nectin 4) [6], as well as CD150 (SLAM, signaling via the lymphocyte activation molecule) [7]. The M protein promotes virus particle formation and maturation, whereas the F protein is associated with membrane fusion [1, 2]. The F protein of MV is initially synthesized as a precursor, F0, which is subsequently cleaved into F1 and F2 by a furin-like protease in the host cell, although they remain linked by a disulfide bond [8]. It also possesses two highly conserved heptad repeat (HR) regions, HR1 and HR2 [9, 10]. After binding to the host cell receptor *via* the H protein, membrane fusion occurs *via* a conformational change in the F protein. Thus, HR1 and HR2 interact to form a six-helix coiled-coil bundle, which is centered on HR1 and surrounded by HR2 [9].

Subacute sclerosing panencephalitis (SSPE) is a rare, but slow viral infection that affects the central nervous system of the fetus, which is caused by persistent MV infection that continues several years after the initial acute infection. It results in extensive cognitive disorders, lapse into vegetative state, and death [11]. Although SSPE is very rare since the widespread introduction of measles vaccines in developed countries [12], it remains an important disease due to its poor prognosis and the absence of an established treatment. Several drugs, such as inosiplex [13, 14] and interferon-α (IFN-α) [15, 16], have been reported to be therapeutic, although they are not curative. Recently, intraventricular ribavirin administration was shown to suppress the spread of the SSPE virus in the central nervous system and block the progression of SSPE syndrome [17–19], although definitive evidence of its clinical efficacy is lacking. Therefore, novel therapeutic strategies are required.

MV strains isolated from patients with SSPE differ virologically from the wild-type MV strain. The SSPE virus has strong neurovirulence and is defective in the production of cell-free infectious virus, which leads to its dissemination via cell-to-cell infection. Autopsy findings of SSPE patients suggested that SSPE virus transmitted via trans-synaptic transmission, and thus, it did not form syncytia [20]. The interaction of F protein with neurokinin-1 triggers a microfusion at the synapse, resulting in trans-synaptic transmission [21]. M protein mutations are associated with these virological characteristics and with SSPE virus pathogenesis [1, 22, 23]. The F protein plays an important role in cell-to-cell infection and is also associated with its neurovirulence [24].

In the case of human immunodeficiency virus type-1 (HIV-1), conformational changes in the envelope glycoprotein gp41, which comprises two α-helical domains—the N-terminal heptad repeat (N-HR) and the C-terminal heptad repeat (C-HR)—have essential roles in the viral fusion process. During fusion, N-HR forms a trimeric coiled-coil surrounded by three C-HRs [25, 26]. For HIV-1, it has been reported that peptides derived from the HR2 of glycoprotein gp41 inhibit viral fusion with the host cell membrane. Some of these peptides, such as enfuvirtide (also referred to as T-20 or DP178 in other studies) have been confirmed to be safe and efficacious and have been clinically utilized as anti-HIV agents for treatment [27]. Recently, some studies have shown that stabilization of the α-helix structure of these peptides has an important role in their antiviral activity. The introduction of a salt bridge formation between two adjacent amino acid residues is one approach for stabilizing the α-helix structure [28–31]. Oishi *et al*. reported that enfuvirtide containing glutamate (E) and lysine (K) substitutions to introduce this salt bridge formation retained stable α-helicity and exhibited highly potent anti-HIV activity [29].

**Fig 1:**
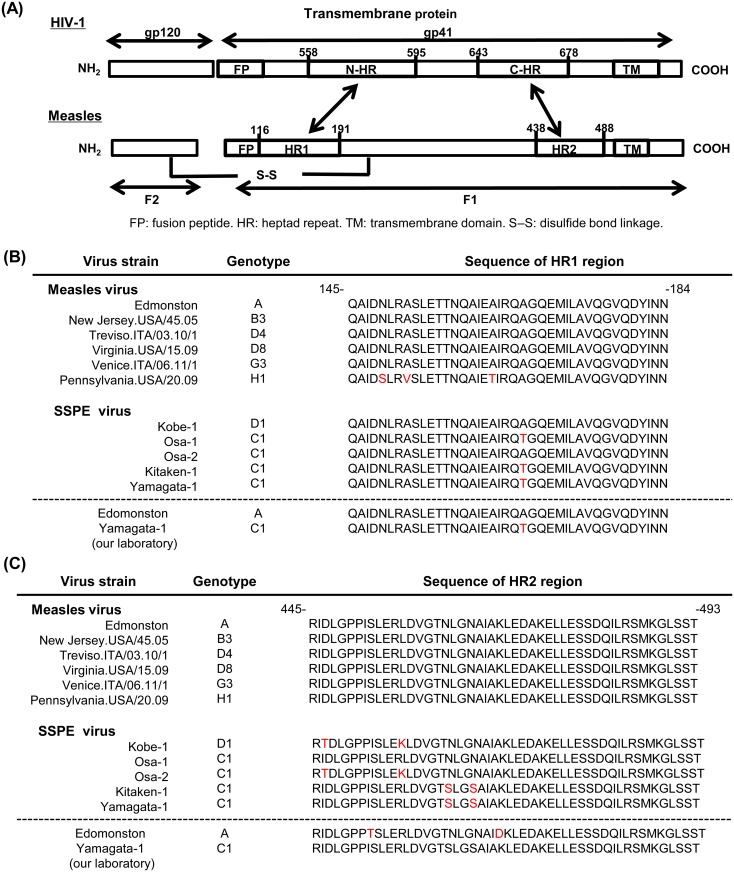
Fusion protein of the measles virus and HIV-1 and amino acid sequences of measles virus heptad repeat (HR) regions. (A) A schematic of the HIV-1 transmembrane protein and measles virus fusion protein showing the locations of structurally significant domains, including the HR region. There are structural and functional similarities between the measles virus and HIV-1 virus. Amino acid sequence data for the measles virus HR1 region (positions 145–184) (B) and the HR2 region (positions 445–493) (C). Amino acid sequence data on measles virus strains were selected based on Measles Surveillance Data of World Health Organization that were currently active measles virus clades worldwide. (http://www.who.int/immunization/monitoring_surveillance/burden/vpd/surveillance_type/active/measles_monthlydata/en/) The letters in red show the amino acid substitution points compared with the Edmonston strain. The amino acid sequences in the lower column represent those of the HR regions of the Edmonston and SSPE Yamagata-1 strains used in our experiments. The amino acid position numbers shown here relate to the MV Edmonston strain sequence, which is available in the UniProtKB/Swiss-Prot database under accession number P69353. The amino acid sequences of other viruses were derived from the GenBank and DDBJ databases under the following accession numbers: New Jersey.USA/45.05, JN635408; Treviso.ITA/03.10/1, KC164757; Virginia.USA/15.09, JN635404.1; Venice.ITA/06.11/1, KC164758; Pennsylvania.USA/20.09, JN635411; Kobe-1, AB254456; OSA-1, AF179433; OSA-2, AF179436; Kitaken, AB453046; and Yamagata-1, D10548.

The structure–function relationships of the fusion protein HR regions are similar in retroviruses and paramyxoviruses (Fig 1A) [10, 32]. Lambert *et al*. identified conserved HR regions within the fusion proteins of paramyxoviruses, including MV, which corresponded to the HR regions of HIV-1 gp41. They also reported that some of the peptides derived from the paramyxovirus fusion protein region had highly selective antiviral activities *in vitro* [10]. Similar results have been reported for overlapping peptides in MV [9, 33], but there are no reports of their antiviral effects in the SSPE virus.

In this study, we synthesized novel peptides derived from the fusion protein HR2 region of MV and evaluated their inhibitory effects on the replication of the measles and SSPE virus *in vitro* and *in vivo*.

## Materials and Methods

### Cells, media, and viruses

African green monkey kidney (Vero) cells and Vero cells expressing human SLAM (Vero/SLAM cells) were used in all experiments. The growth medium for Vero cells comprised minimal essential medium (MEM) supplemented with 10% heat-inactivated newborn bovine serum, penicillin G (100 IU/ml), streptomycin (100 μg/ml), and l-glutamine (300 μg/ml). The same growth medium, which was further supplemented with G418 (400 μg/ml), was used for Vero/SLAM cells. Cells were seeded into the wells (22 mm) of 12-well tissue culture plates at 1 × 10^5^ cells/well and incubated at 37°C in a 10% CO_2_ incubator for 3 days, at which time they had just reached confluence. The virus strains used were human MV Edmonston strain and SSPE Yamagata-1 [34], which was originally isolated from the brain of an SSPE patient. The Edmonston strain of MV was used to inoculate Vero/SLAM cells and harvested after freeze–thawing infected cells. The SSPE Yamagata-1 strain was cultured in Vero/SLAM cells, harvested by trypsinization, prepared as an infected cell suspension in the Vero/SLAM cell medium supplemented with 10% dimethyl sulfoxide, and stored at −80°C until use.

### Gene sequence analysis

Viral RNA was extracted from the MV Edmonston and SSPE Yamagata-1 strains using ISOGEN-LS (Nippon Gene, Tokyo, Japan). After RNA extraction, cDNA was synthesized using a reverse transcription kit (PrimeScript RT-PCR kit, TaKaRa, Shiga, Japan), and PCR was performed using TaKaRa Ex Taq^®^ HS according to the manufacturer’s instructions. Primers targeting the coding regions of F genes were synthesized, as previously described [35]. PCR products were separated by 2% agarose gel electrophoresis, and the bands of interest were excised. DNA was purified using a QIAquick Gel Extraction Kit (Qiagen, Hilden, Germany), and the direct sequence analysis of F genes was performed using a DYEnamic ET Terminator Cycle Sequencing Kit (GE Healthcare, Tokyo, Japan). Sequence data were then analyzed using GENETYX Ver. 10 (Genetyx Corporation, Tokyo, Japan). The amino acid sequence was deduced from the nucleotide sequence using the GENETYX software.

### Peptide synthesis

**Fig 2:**
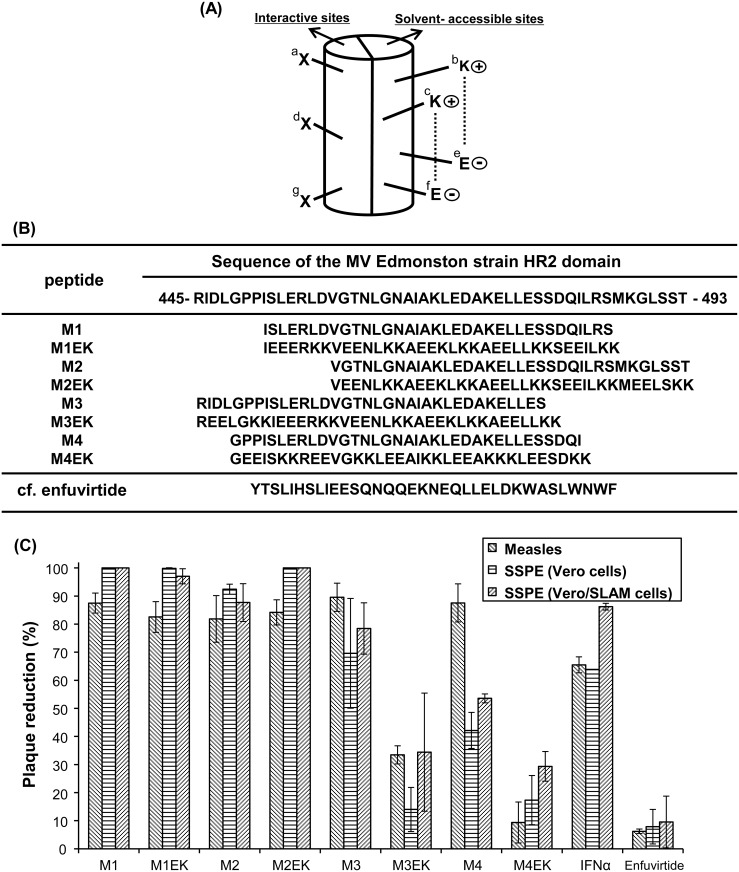
Peptide synthesis and screening. (A) The preordered α-helix structure of the synthetic peptide with salt bridge formation. Glutamate (E) and lysine (K) were introduced into solvent-accessible sites (the e/f and b/c positions, respectively) to induce salt bridge formation, while maintaining the amino acids in the interactive sites, thereby resulting in a repeat of X-EE-XX-KK (X indicates the original amino acid) motifs. (B) Amino acid sequences of synthetic peptides derived from the MV Edmonston strain HR2 domain. M1EK, M2EK, M3EK, and M4EK, in which the α-helix structure is stabilized by the formation of a salt bridge, are synthetic peptides based on peptides M1, M2, M3, and M4, respectively. Enfuvirtide is a peptide fusion inhibitor of HIV-1. (C) Screening of synthetic peptide data. The vertical axis represents plaque reduction as a percentage relative to the control group. Peptide screening was performed at a concentration of 10 μM. Enfuvirtide and interferon-α (IFN-α) were used as a control peptide at a concentration of 10 μM and 250 IU/ml, respectively. The experiments were performed in duplicate, and at least twice independently with similar results. Error bars indicate the standard deviation.

Eight peptides derived from the HR2 region based on the amino acid sequence data for the MV Edmonston strain were synthesized. M1, M3, and M4, which are peptides with antiviral activity against the MV Edmonston strain, were synthesized, as previously reported [10]. M2 is a novel peptide with the same length (35 amino acids) as that of M1, M3, and M4. Peptides with an EK motif, i.e., M1EK, M2EK, M3EK, and M4EK, were also novel synthetic peptides that possessed α-helix structures stabilized by the formation of a salt bridge. Salt bridge formation was induced by replacing amino acids at solvent-accessible sites of the helical bundle with glutamate (E) and lysine (K), while maintaining those at the interactive sites because these are critical for interactions with the HR1 region. E and K were arranged at the e/f and b/c positions, respectively (Fig 2A), yielding a repeat of X-EE-XX-KK (X indicates the original amino acid) motifs.

All peptides were synthesized as N-terminally acetylated and C-terminally amidated forms using standard 9-fluorenylmethoxy carbonyl-based solid-phase techniques [30]. Peptide samples were prepared for biological tests by high-performance liquid chromatographic purification of crude materials on a preparative Cosmosil 5C18 AR-II column (Nacalai Tesque Inc., Kyoto, Japan) using a linear gradient of H_2_O/acetonitrile containing 0.1% trifluoracetic acid. Enfuvirtide was purchased from Genentech Inc. and Trimeris Inc., (San Francisco, CA, USA). The amino acid sequence of enfuvirtide is as follows: YTSLIHSLIEESQNQQEKNEQLLELDKWASLWNWF.

### Screening and 50% effective concentration determination

Screening was performed to evaluate the antiviral effects of each synthetic peptide against the MV and SSPE viruses using the viral plaque reduction assay. Based on the results of the peptide screening, the 50% effective concentration (EC_50_), i.e., the concentration of compounds required to inhibit virus plaque formation by 50%, was also determined. The Human MV Edmonston strain was used to produce cell-free virions, which yielded a reproducible number of syncytia [generally 200–300 plaque-forming units (PFUs)] in the control wells. The SSPE virus was also used to yield a reproducible number of syncytia (generally 70–150 PFU) in control wells. In the peptide screening assay, each synthetic peptide was used at a concentration of 10 μM. IFN-α and enfuvirtide, a peptide fusion inhibitor of HIV-1, were used as controls at a concentration of 250 IU/ml and 10 μM, respectively.

### Cytotoxicity assays

Peptide cytotoxicity was determined using the methyl tetrazolium assay. Five-fold serial dilutions of peptides were added to confluent Vero or Vero/SLAM cells. Cells were then incubated at 37°C for 24 h, and the number of viable cells was determined using 3-(4,5-dimethyllthiazol-2-yl)-2,5-diphenylterazolium bromide (Sigma–Aldrich, Tokyo, Japan). The 50% cytotoxic concentration (CC_50_), which was defined as the concentration required to reduce the cell viability by 50% relative to that of untreated controls, was determined. The selectivity index (SI) was defined as the ratio of the CC_50_ relative to the EC_50_ value.

### Time-of-addition experiments

Synthetic peptides, M1 and M2EK (10 μM each) were used in time-of-addition experiments. Each peptide was added to Vero cells at three distinct time points: prior to infection (pretreatment with the compound), at the same time as the virus infection (simultaneous infection), or post-infection (post-entry). The SSPE virus was prepared to yield a reproducible number of syncytia in untreated control wells (approximately 50–100/well). In the pretreatment assay, peptide was added to confluent cells and incubated at 37°C for 30 min, and the cell cultures were washed with MEM before viral inoculation. Cells were then challenged with the SSPE virus at 0°C for 30 min. In the simultaneous infection assay, SSPE virus with peptide was inoculated at 0°C for 30 min. After the adsorption, the cell cultures were subsequently washed with MEM, and growth medium containing 0.75% methylcellulose was overlaid on the cell cultures, followed by incubation for 3 days in both pretreatment and simultaneous infection assays. In the post-entry assay, cells were challenged with the SSPE virus at 0°C for 30 min. After adsorption, the cell cultures were subsequently washed with MEM and peptide, which was prepared in growth medium containing 0.75% methylcellulose, was subsequently overlaid on the cell cultures at distinct time points. The starting point when cells were incubated at 0°C for 30 min was defined as t0.

### Animal survival experiments

Based on a previous report [36], but with slight modifications, 3-week-old female BALB/cAJcl-nu/nu mice (CLEA Japan Inc., Tokyo, Japan) were used in the animal experiments. Mice were divided in four groups (n = 6 for each cage) and housed with free access to both water and food and monitored daily. The mice were SSPE virus-infected or mock-infected (MEM alone) intracranially. An SSPE virus-infected cell suspension containing 250 PFU/ 30 μl (100% lethal dose) was inoculated into the subarachnoid space of nude mice using a 27-gauge double needle at a depth of 2 mm. Each compound was prepared at a concentration of 100 μM and was injected intracranially at 0 (mixed with viruses), 6, and 24 h after the initial virus inoculation. All injections were performed under inhalational anesthesia with isoflurane. Each mouse was checked for neurological symptoms and weighed daily. The ethical endpoint was defined as <75% of their maximum weight because most mice would die within 24 h. On attaining this endpoint, the mice were sacrificed by cervical dislocation under inhalational anesthesia with isoflurane. In this experiment, five mice died of natural causes before the ethical endpoint; however, all mice had neurological symptoms and 9.5%, 13.2%,14.5%, 15.8%, 16.4% weight loss (one each) relative to their maximum weight. This study was approved by the control of the Animal Research Committee in accordance with the Guidelines on Animal Experiments in Fukushima Medical University and the Rules for the Protection and Care of Animals (approval number: 23038).

### Viral quantification

Mice were divided in three groups (n = 5 for each cage) and housed with free access to both water and food and monitored daily. Mice were infected with SSPE virus intracranially in the same as animal survival experiments. Euthanasia was performed by cervical dislocation under isoflurane anesthesia on day 14 post-infection. The brain of each mouse was immediately harvested and mechanically homogenized with 2 ml Vero/SLAM cell growth medium using a PRO200 handheld or post-mounted homogenizer (PRO Scientific Inc., CT, USA). The homogenized brains were stored at −80°C. Viral RNA was extracted using ISOGEN-LS, and cDNA was synthesized using a reverse transcription kit (PrimeScript RT-reagent kit). MV RNA was quantified using a TaqMan real-time polymerase chain reaction assay, as previously described [37, 38]. The primer and probe were designed based on the MV Edmonston strain N gene, as previously reported [39]. PCR amplification was performed using a 7300 Real-Time PCR system (Applied Biosystem). The control plasmid was produced by Dragon Genomics Center (Takara Bio, Mie, Japan) based on the MV Edmonston strain N gene (position 1321–1500).

### Statistical analysis

All values are expressed as the mean ± standard deviation (SD). Comparisons among groups of peptide screening data were made using one-way analysis of variance (ANOVA), followed by Dunnett’s post-hoc test. Differences between groups were analyzed using a log rank test with Tukey’s post-hoc test (for mortality data) or Kruskal–Wallis test with Steel-Dwass post-hoc test (for time-of-addition date and viral RNA data). Statistical analyses were performed using Excel Statistics 2012, Social Survey Research Information Co. Ltd., Tokyo, Japan. *P* < 0.05 was considered statistically significant.

## Results

### Amino acid sequences of the MV Edmonston and SSPE Yamagata-1 strains

The amino acid sequences of the HR1 regions between strains were the same, or had only one substitution, except for that of Pennsylvania.USA/20.09 strain. By contrast, zero or two amino acid substitutions were detected between virus strains in the HR2 region (Fig 1B and 1C). These results suggest that HR regions of the F protein are comparatively well conserved between virus strains, particularly the HR1 region. Moreover, it has been shown that synthetic peptides derived from the HR2 region alone could inhibit MV viral fusion [10, 33]. Thus, we synthesized peptides derived from the HR2 region based on the amino acid sequence data for the MV Edmonston strain. To compare the virus strains in this study with those in previous reports, amino acid sequences of HR regions of the SSPE Yamagata-1 strain were identical to those previously described [40], whereas two different amino acid sequences were identified in the HR2 region of MV Edmonston strain (Fig 1B and 1C).

### Peptide screening and *in vitro* efficacy of the novel peptide

Eight peptides were synthesized based on the amino acid sequence data for the HR2 region of the MV Edmonston strain (Fig 2B). The eight synthetic peptides were screened at a concentration of 10 μM. The novel peptides, except for M4EK, showed statistically significant inhibition of MV Edmonston plaque formation compared with the control group (*P* < 0.01; Dunnett’s post-hoc test) (Fig 2C). In contrast, plaque numbers among all groups were significantly different from the SSPE Yamagata-1 strain in both Vero cells (*P* < 0.01; ANOVA, *F* = 64.9) and Vero/SLAM cells (*P* < 0.01; ANOVA, *F* = 47.4). In particular, M1, M1EK, and M2EK inhibited plaque formation by >97% compared with the control group (*P* < 0.01; Dunnett’s post-hoc test) (Fig 2C). M3EK, M4EK and Enfuvitide had lower antiviral activity against both Edmonston and Yamagata-1 strains than the other peptides (Fig 2C).

Following the results of the peptide screening, we determined EC_50_ values of M1, M1EK, M2, and M2EK against the MV Edmonston strain (Table 1). M1, M1EK, M2, and M2EK had similar antiviral activity levels against the Edmonston strain.

**Table 1:** Effects of synthesized peptides on the measles and SSPE viruses.

	Measles virus (Vero cells)			SSPE virus (Vero cells)			SSPE virus (Vero/SLAM cells)		
Peptide	EC_50_^a^ (μM)	CC_50_^b^ (μM)	SI^c^	EC_50_^a^ (μM)	CC_50_^b^ (μM)	SI^c^	EC_50_^a^ (μM)	CC_50_^b^ (μM)	SI^c^
M1	0.03 ± 0.01	>100	>3663	0.01 ± 0.01	>100	>7519	0.01 ± 0.01	>100	>9524
M2	0.12 ± 0.04	>100	>805	0.26 ± 0.17	>100	>377	0.48 ± 0.22	>100	>209
M2EK	0.10 ± 0.07	>100	>989	0.05 ± 0.03	>100	>2041	0.02 ± 0.01	>100	>6579
Enfuvirtide	>10	>100	-	>10	>100	-	>10	>100	-

^a^EC_50_, 50% effective concentration or the concentration required to reduce cell viability by 50%.

^b^CC_50_, 50% cytotoxic concentration or the concentration required to inhibit viral replication by 50%.

^c^SI, Selectivity index is the ratio of the 50% effective concentration to the 50% cytotoxic concentration and indicates drug tolerability *in vitro*.

The EC_50_ values of M1, M1EK, M2, and M2EK against the Yamagata-1 strain were 0.01 ± 0.01, 0.12 ± 0.05, 0.26 ± 0.17, and 0.05 ± 0.03 (μM; mean ± SD) (Table 1), respectively, in Vero cells and 0.01 ± 0.01, 0.17 ± 0.02, 0.48 ± 0.22, and 0.02 ± 0.01 (μM; mean ± SD) (Table 1), respectively, in Vero/SLAM cells. M1 and M2EK had almost the same antiviral activity levels against the SSPE Yamagata-1 strain. Enfuvirtide, the control peptide derived from the HR2 of HIV-1 gp41, had no activity against Edmonston and Yamagata-1 strains at concentrations up to 10 μM. The CC_50_ values were >100 μM for all peptides in both Vero and Vero/SLAM cells, and none of the synthetic peptides had any cytotoxic effect. Therefore, all peptides had SI values in the order of several hundreds to several thousands (Table 1).

### Antiviral activity mechanism of the peptide *in vitro*

**Fig 3:**
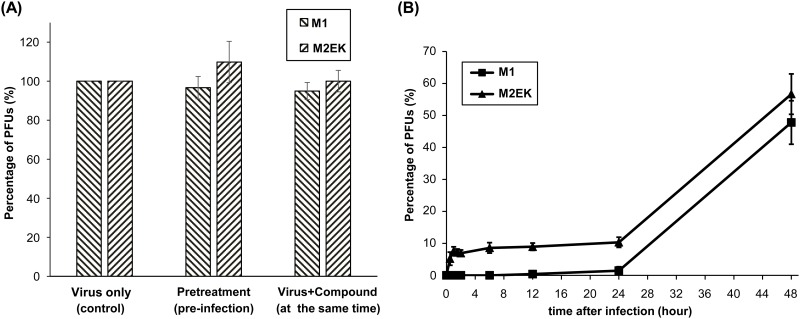
Effects of the novel peptide on PFU at different stages of virus infection. The synthetic peptides M1 and M2EK were added at three distinct time points: prior to infection (pretreatment), at the same time as virus infection, or post-infection (post-entry). (A) Effects of each peptide administration prior to or at the same time as virus infection: There was no statistically significant difference in viral plaque numbers between groups or peptides. The experiments were performed in duplicate, three times independently and error bars represent the standard deviation of the means. (B) Effect of each peptide administration post-infection: The starting point when cells were incubated at 0°C for 30 min was defined as t0. The vertical axis represents the percentage of plaque forming units (PFUs) for the control group in both figure panels. Both peptide inhibited viral replication during the viral fusion stage but not during the adsorption stage. The error bars indicate the standard deviation of three independent experiments, each performed in duplicate.

Time-of-addition experiments were performed to determine the stage at which compounds exerted their inhibitory effects using the synthetic peptides M1 and M2EK. When each peptide was added before viral adsorption or at the same time as virus inoculation, no reduction in viral plaque numbers was detected compared with that in each control group (Fig 3A). Moreover, there were no statistically significant differences between peptides at pretreatment or when added at the same time as virus infection (Fig 3A). These results suggest that the novel peptides did not inhibit infection at the adsorption stage. Based on these results, we added each peptide to cell cultures at distinct time points after viral adsorption. Vero cells were challenged with the virus and incubated at 0°C; therefore, the virus remained at the attachment stage and did not progress to the fusion stage. In this assay, the viral inhibitory effect of each peptide was observed in a time-dependent manner, particularly within 24 h (Fig 3B). This suggests that both peptides interfere with viral fusion stage, resulting in the reduction of viral replication.

### *In vivo* effects of the novel peptides

**Fig 4:**
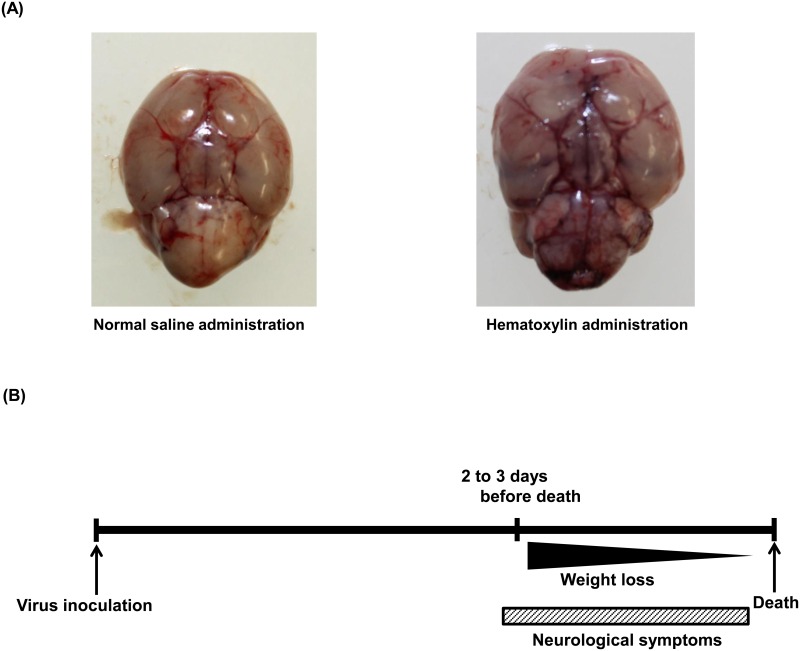
Time course of SSPE virus-infected nude mouse model. (A) The brains of nude mice were harvested 3 h after 30 μl intracranial hematoxylin or normal saline administration. Hematoxylin was found to have spread beneath the subarachnoid space. (B) Nude mice exhibited weight loss 2–3 days before death and died 16–31 days after virus inoculation. Neurological symptoms were observed shortly before or at the same time as weight loss began; typical neurological symptoms in nude mice were sudden jumps, convulsions, hypersensibility, and gait disorder, but the degree of these symptoms was different for each mouse.

To confirm whether the compound or virus had spread beneath the subarachnoid space of the nude mice, 30μl hematoxylin was intracranially administered. Hematoxylin was found to have spread beneath the entire subarachnoid space within 3 h of administration (Fig 4A). Based on a previous report with slight modifications [36], we established the SSPE virus-infected nude mouse model. In this model, symptoms such as sudden jumps, hypersensibility, convulsions, and gait disorders were observed during SSPE development. The nude mice then exhibited progressive weight loss, gradual loss of mobility, and finally death (Fig 4B).

**Fig 5:**
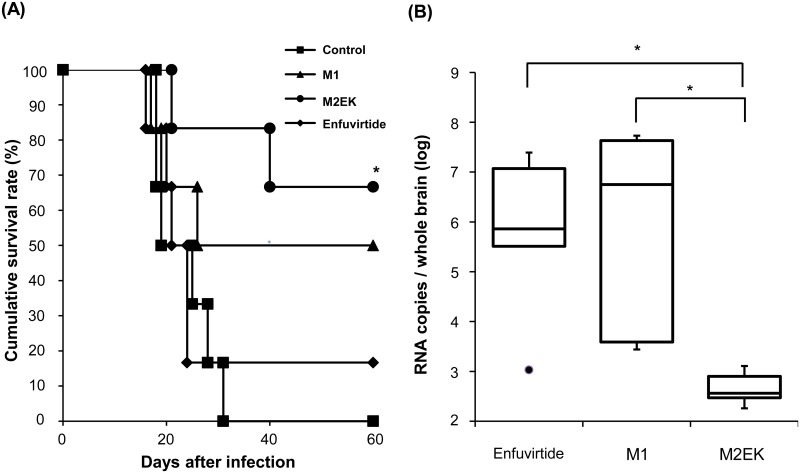
Effects of synthesized peptides on the SSPE virus in nude mice. (A) 24 nude mice (n = 6 for per group) were intracranially infected with the SSPE Yamagata-1 strain and administered M1, M2EK, or enfuvirtide. Control mice were administered minimal essential medium alone. The difference in the survival rate of mice receiving M2EK was statistically significant compared with that of control mice, whereas there was no statistically significant difference in survival rate between other pairwise groups (*P* < 0.05, log rank test for survival curves; post-hoc comparisons with Tukey’s test). (B) 15 nude mice (n = 5 per group) were intracranially infected with the SSPE Yamagata-1 strain and administered M1, M2EK, or enfuvirtide. Viral RNA copies in each entire brain were quantified on day 14 post-infection. Error bars indicate the standard error of the mean. *P < 0.05; Kruskal–Wallis test, Steel–Dwass post-hoc test.

M1 and M2EK, both of which exhibited antiviral efficacy *in vitro*, were intracranially administered in a nude mouse model to evaluate their therapeutic efficacy and toxic effect. Enfuvirtide was used as a control peptide. In the control group, all nude mice died or required euthanasia due to weight loss (range = 18–31 days post-infection). Intracranial administration of M2EK in nude mice increased the survival rate from 0% to 67% compared with the control group (*P* < 0.05). By contrast, there was no statistically significant difference in survival rate between other pairwise groups (Fig 5A).

To further evaluate the inhibitory effect of M2EK, we determined the viral RNA copy number in whole brains on day 14 post-infection. The viral RNA copies in M2EK-treated brains were approximately four log lower than in the brains of M1-treated mice (*P* < 0.05), and approximately three log lower than in the brains of enfuvirtide-treated mice (*P* < 0.05) (Fig 5B). These results suggest that M2EK spreads to the cerebral parenchyma *via* the subarachnoid space and markedly inhibits the replication of the SSPE virus, thereby leading to an increased survival rate.

Mice administered with peptides alone (M1, M2EK, and Enfuvirtide at the concentration of 100 μM each) survived for >45 days with no symptoms, indicating that these peptides show no toxicity *in vivo*.

## Discussion

To our knowledge, this is the first study to demonstrate that novel synthetic peptides derived from the HR2 region of MV inhibit the replication of the SSPE virus *in vitro* and *in vivo*.

Inhibition of the fusion process is an effective strategy for SSPE treatment because the SSPE virus is characteristically defective in the production of cell-free infectious virus, which leads to its dissemination *via* cell-to-cell fusion. In this study, we demonstrated that synthetic peptides inhibited the replication of the MV and SSPE viruses at nanomolar concentrations without any cytotoxicity (Table 1). In addition, our study showed that the peptides inhibited viral replication by interfering with the fusion process (Fig 3).

The synthetic peptides had antiviral activity against the SSPE virus despite the fact that they were derived from the MV Edmonston strain. During the viral fusion process, conformational changes in the F protein that occur *via* interactions between HR1 and HR2 regions, to form a stable six-helix coiled-coil bundle, are essential in paramyxoviruses. Several studies have shown that the HR regions of the F protein are highly conserved in paramyxoviruses [41, 42]. Moreover, the highly conserved region is critical for correct F protein folding during the fusion process [42]. These facts indicate that HR regions of the F protein are essential components; thus, any major mutation in these regions may be fatal for the virus because they could reduce its viral replication capability and/or infectivity. In our study, one or two amino acid substitutions were observed in HR regions of the Edmonston and Yamagata-1 strains (Fig 1B and 1C). Komase *et al*. reported that the amino acid sequence of the MV F protein shared 96.27% homology with the SSPE Yamagata-1 strain [40]. This supports the fact that amino acid sequences of HR regions of the SSPE virus, an MV variant, are well conserved compared with MV; therefore, synthetic peptides derived from HR regions of the MV F protein also had antiviral activity against the SSPE virus.

We also observed that M2EK, which is formed through the introduction of an EK motif into M2, exhibited higher antiviral activity against the MV and SSPE viruses than does M2 *in vitro*. These results suggest that the introduction of the EK motif into the HR2 peptide increased its binding affinity to the HR1 region of the MV fusion protein. Several reports have shown that introduction of EK substitution to synthetic peptides at solvent-accessible sites retain an α-helical conformation and increased the binding affinity for target the HR1 regions of wild-type and mutant viruses [29, 31, 43]. It is possible that M2EK possesses these physicochemical properties, but not M2. However, the other three peptides with the EK motif, i.e., M1EK, M3EK, and M4EK, had lower antiviral activities than did each parent peptide. EK substitutions in these peptides may induce critical conformational changes, thereby resulting in a low binding affinity or altering contacts to the HR1 region.

M1 and M2EK had almost the same antiviral activity levels against the SSPE Yamagata-1 strain at nanomolar concentrations *in vitro*, but only M2EK administration increased the survival rate and inhibited viral replication in the nude mouse model (Fig 5). In the *in* vivo assay, the thermal stability of peptides would be important for antiviral activity. Naito *et al*. reported that synthetic peptides with an EK substitution exhibit sufficiently high thermal stability to target the HR1 region, which results in highly potent antiviral activities against wild-type and mutant viruses that are resistant to those without the EK motif [31]. There are no previous reports on pharmacokinetic and pharmacodynamic differences *in vivo* between synthetic peptides with or without EK substitution, but differences in these pharmacokinetic and pharmacodynamic characteristics, such as the metabolism rate of peptides, may contribute to differences in the inhibition of viral replication *in vivo*.

In summary, the novel peptide M2EK, which was derived from the HR2 region, inhibited the replication of the SSPE virus by interfering with its fusion process *in vitro* and *in vivo*. The inhibition of the viral fusion process during infection may be a useful strategy for the treatment of incurable diseases, such as SSPE. Thus, M2EK may be a new candidate for SSPE treatment.
